# A Specimen-Separated Machine Learning Benchmark Toward Real-Time Tissue-Type Identification in Guided Surgery Using Ex Vivo Bovine Laser-Induced Breakdown Spectroscopy

**DOI:** 10.3390/s26165020

**Published:** 2026-08-07

**Authors:** René Fernando Sosa-Santos, José Luis Arce-Diego, Félix Fanjul-Vélez

**Affiliations:** 1 Biomedical Engineering Group, TEISA Department, Universidad de Cantabria, 39005 Santander, Spain; rss639@alumnos.unican.es (R.F.S.-S.); arcedj@unican.es (J.L.A.-D.); 2 Optical and Energy Research Center, Universidad Privada Boliviana, Cochabamba, Bolivia

**Keywords:** laser-induced breakdown spectroscopy, tissue classification, guided surgery, intraoperative tissue identification, machine learning, group-based cross-validation, data leakage, random forest, extra trees, surgical margin assessment

## Abstract

Real-time tissue identification during laser-guided surgery is a critical unmet need for collateral damage avoidance and margin delineation. Laser-Induced Breakdown Spectroscopy (LIBS) is compatible with pulsed laser surgical systems and offers rapid, label-free elemental analysis. This study presents a machine learning pipeline classifying five ex vivo bovine tissue classes, plus one synthetic null-signal control class, from LIBS spectra, designed to control specimen-level data leakage and class imbalance bias. Key contributions are (i) a ‘peak max over baseline’ aggregation strategy suppressing shot noise while preserving emission peaks; (ii) a repeated, group-based cross-validation protocol (GroupShuffleSplit, *N* = 10) enforcing specimen-level separation; and (iii) a comparison of 30 configurations (10 classifiers × 3 pipelines). Extra Trees with normalization reached the highest weighted F1-score (0.934 ± 0.118); excluding the synthetic control, five-class scores fall to 0.875–0.915 and the ranking changes, so these are the reference figures for biological tissue discrimination. Support Vector Machines were less accurate but more consistent (0.917 ± 0.069). Acquisition takes approximately 3 s per point; inference is sub-millisecond. With five source animals, the best configuration chosen on the same outer splits, and inner tuning that was not group-aware, these estimates are an exploratory step toward real-time guided surgery.

## 1. Introduction

Precise identification of tissue type and boundary delineation are critical determinants of success in numerous surgical interventions, spanning oncological resection, nerve-sparing procedures, and minimally invasive guided surgery. The status of surgical margins, whether free of tumor cells or positive, is among the strongest predictors of long-term clinical outcome, with positive margins directly associated with elevated rates of local recurrence and reduced patient survival [1,2]. The current intraoperative standard, frozen-section histopathology, requires tissue excision, cryosectioning, and expert pathological interpretation, a process that is inherently resource-intensive, prolongs anesthesia duration and remains susceptible to sampling error and inter-observer variability [3,4]. Crucially, this approach provides only discrete, static snapshots of tissue status and cannot continuously guide the surgical instrument at the ablation interface. There is a compelling, unmet clinical need for diagnostic technologies capable of providing accurate, in situ, and real-time feedback to support intraoperative decision-making.

Laser-Induced Breakdown Spectroscopy (LIBS) is uniquely positioned to address this need. As an atomic emission spectroscopy technique, LIBS focuses a high-energy, nanosecond laser pulse onto the tissue surface, generating a micro-plasma whose characteristic emission spectrum encodes the elemental composition of the ablated material [5]. This process is inherently compatible with laser surgical systems: the same focused beam employed for tissue ablation or incision can serve simultaneously as the excitation source for spectroscopic analysis, enabling seamless integration without additional hardware or sample preparation. Key analytical attributes, near-instantaneous analysis time (below 1.05 ms per laser pulse), no reagents, and compatibility with fiber-optic probes for endoscopic or laparoscopic delivery, make LIBS uniquely suited for real-time, in situ tissue identification during guided surgical procedures [6,7]. Multiple studies have successfully demonstrated the feasibility of LIBS for differentiating biological tissues in ex vivo and in vivo settings by exploiting tissue-specific elemental fingerprints, including discrimination of liver tumors, lung tissue, and human electrolyte profiles in various tissue matrices [8,9,10,11].

Within the broader landscape of optical tissue characterization for guided surgery, complementary spectroscopic and imaging modalities have also demonstrated promising tissue discrimination capabilities: diffuse reflectance spectroscopy has been combined with machine learning classifiers for ex vivo biological tissue characterization [12,13], fluorescence spectroscopy has been investigated for nerve-specific biomarker detection in surgical guidance applications [14], and artificial intelligence-assisted phase-contrast digital histopathology has shown feasibility for label-free tumoral tissue discrimination [15,16]. These parallel developments reinforce the concept of label-free optical tissue identification as a viable paradigm for guided surgery and motivate the development of rigorous, reproducible machine learning frameworks applicable across spectral modalities.

Despite its considerable analytical potential, translating raw LIBS spectra into robust, clinically deployable machine learning classifiers presents substantial methodological challenges. First, LIBS spectra are inherently high-dimensional: each spectrum encodes intensity information across thousands of discrete wavelength channels, creating a severe *p* ≫ *n* scenario that greatly elevates the risk of model overfitting [17,18]. Second, and more critically, biomedical spectral datasets exhibit a nested hierarchical structure: tens to hundreds of spectra are acquired from each biological specimen. When this hierarchy is ignored during cross-validation, a common practice in the literature, spectra from the same specimen can appear in both the training and test sets, allowing the model to memorize specimen-specific signatures. This serious methodological pitfall, known as data leakage, produces inflated, non-reproducible performance estimates that do not reflect true generalization to unseen specimens [19]. Third, natural variation in specimen counts per tissue type creates class imbalance, which can bias standard classifiers toward the majority class and yield misleadingly high aggregate accuracy metrics.

A review of the existing literature on LIBS-based tissue classification reveals that near-perfect classification rates are frequently reported: accuracies above 95% and up to 100% for hepatic tissue discrimination [10] and 98.9% for lung tissue [11], in both cases estimated by random cross-validation at the spectrum level, without separating specimens by source animal or patient. Such results are often artifacts of the methodological shortcomings described above—particularly data leakage arising from the failure to enforce specimen-level data separation during cross-validation—rather than genuine indicators of model generalizability [5,19]. Specifically, when multiple spectra from the same specimen are distributed across the training and test sets, the classifier effectively memorizes specimen-specific spectral signatures, yielding inflated accuracy estimates that do not generalize to unseen specimens. This reproducibility gap impedes the development of clinically reliable diagnostic tools and delays the path toward regulatory approval. This study directly addresses these issues by presenting a rigorous, end-to-end machine learning pipeline explicitly designed for LIBS-based tissue classification. Our specific contributions are as follows: (i) we introduce a ‘peak max over baseline’ non-linear spectral aggregation strategy that suppresses stochastic shot noise while selectively preserving true atomic emission peaks; (ii) we implement a repeated, group-based cross-validation protocol (GroupShuffleSplit, *N* = 10) that enforces specimen-level separation between training and test sets and provides performance estimates with quantified uncertainty; (iii) we conduct a systematic comparison of ten classification algorithms under three distinct feature preprocessing regimes, totaling 30 experimental configurations; and (iv) we critically analyze the performance versus stability trade-off among top-performing models, a dimension of direct importance for clinical deployment that is frequently overlooked in comparative studies. The result is not only a high-performing classification system but, more importantly, a transparent and reproducible performance benchmark serving as a methodological reference for the development of LIBS-based real-time tissue identification systems for guided surgery.

The remainder of this paper is organized as follows. Section 2 describes the biological sample preparation protocol, the custom-built LIBS experimental setup, the multi-step spectral preprocessing and aggregation pipeline, and the rigorous group-based cross-validation framework designed to prevent data leakage. Section 3 presents the comparative classification performance across all 30 experimental configurations, including a detailed analysis of model stability across the 10 repeated outer test evaluations. Section 4 discusses the implications of the results from a biomedical engineering perspective, addressing the role of spectral preprocessing in tissue discrimination, the performance-versus-reliability trade-off in the context of clinical translation, and the critical importance of specimen-level separation in validation as a methodological standard. Finally, Section 5 summarizes the key findings and outlines the most promising directions for future work, including prospective intraoperative validation and the exploration of deep learning architectures for real-time tissue identification.

## 2. Materials and Methods

### 2.1. Sample Preparation and Data Acquisition

Biological tissue samples were sourced from ex vivo bovine specimens obtained immediately following slaughter at a licensed local abattoir. This rapid collection protocol was implemented to minimize post-mortem autolytic and putrefactive changes that could alter the tissue’s intrinsic elemental composition. All samples were transported to the laboratory on the day of slaughter and wrapped in food-grade butcher paper to prevent cross-contamination. Samples not analysed on the collection day were stored refrigerated (approximately 4 °C) in the same wrapping, without immersion in any liquid medium, to preserve the native elemental composition of the tissue surface. No additional chemical preparation was applied prior to LIBS measurement. All LIBS analyses were completed within a three-day post-mortem window to ensure the elemental integrity and consistency of the entire dataset. The specimens analysed in this study were obtained from five bovine animals, collected during two visits to the abattoir (two animals in the first visit and three in the second) in order to work with fresh tissue.

The dataset comprises 69 units (henceforth referred to as ‘groups’ to emphasize their role in the validation protocol): 54 biological specimens collected across five tissue classes—Aorta, Small Intestine, Large Intestine, Connective Muscle and Nerve—together with 15 records of a sixth, non-biological control class, Non-Tissue. The Non-Tissue class is not measured data. It consists of 15 synthetic records, each built by drawing Gaussian noise (mean 100 counts, standard deviation 20) at every wavelength of the instrument axis, so as to emulate an acquisition in which the laser fires but no plasma is formed. Its single purpose was to train the classifier to recognize a null-signal state, a capability required in any deployment scenario in which the beam may fire without ablating tissue. Because these records contain no emission features by construction, they are separable from biological tissue by construction as well; the five-class results reported in Section 3.2, which exclude this class entirely, are therefore the reference figures for discrimination among biological tissues. The distribution of specimens across tissue classes is summarized in Table 1.

Table 1 summarizes the distribution across the five biological tissue classes and the synthetic control class. The dataset presents a pronounced class imbalance: Connective Muscle contributes the largest group (*n* = 16 specimens), while Small Intestine is the most under-represented class (*n* = 6). The synthetic Non-Tissue control class (*n* = 15, Section 2.1) was sized to be comparable to the biological classes. This imbalance is addressed during model training by SMOTE oversampling (Section 2.4.3) and is accounted for by reporting weighted F1-scores throughout.

### 2.2. LIBS Experimental Setup

**Figure 1:**
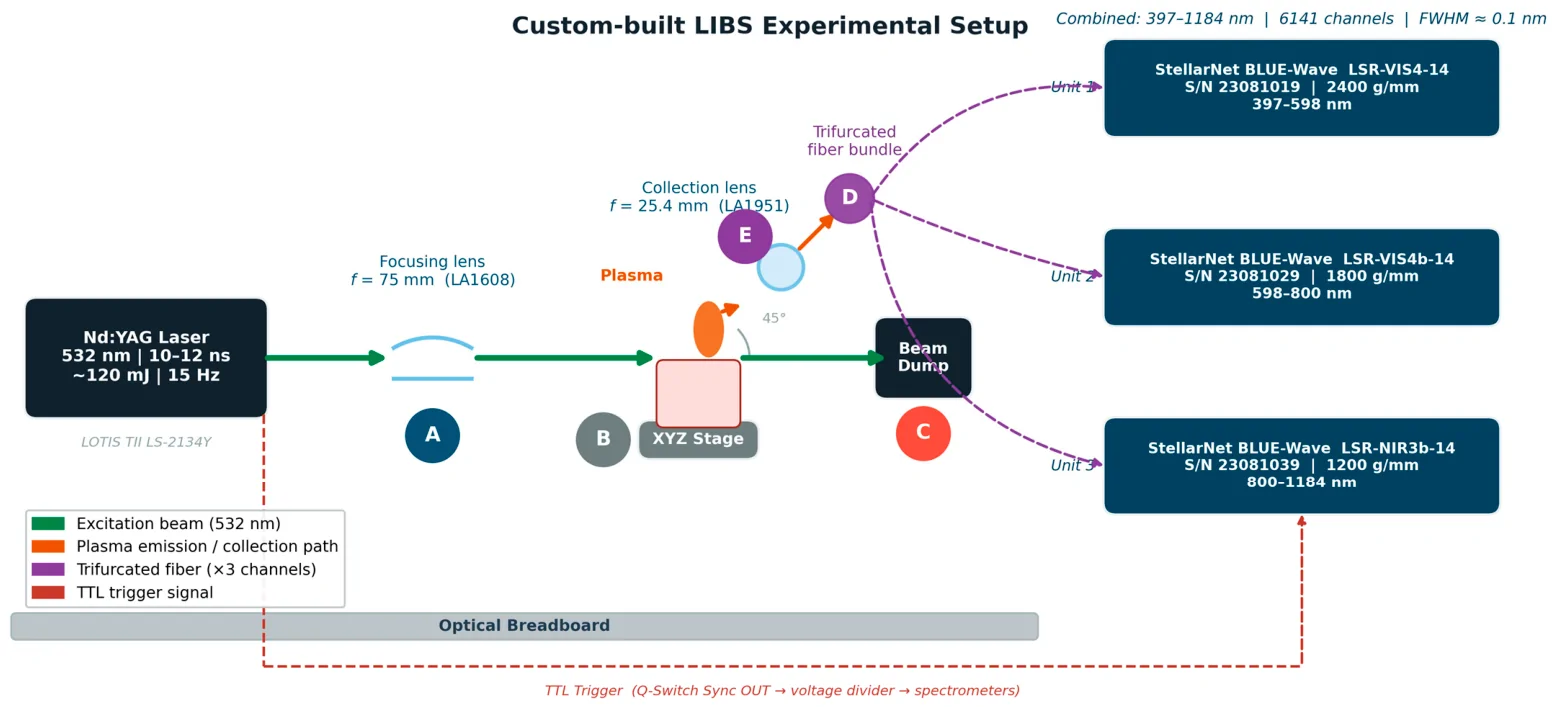
Schematic diagram of the custom-built LIBS experimental setup. The pulsed Nd:YAG laser (532 nm, 10–12 ns, ~120 mJ, 15 Hz) directs the excitation beam through a plano-convex focusing lens (A, f = 75 mm) onto the tissue sample mounted on a manual XYZ positioning stage (B). The transmitted beam is safely terminated by a beam dump (C). Plasma emission is collected at 45° relative to the incident beam by a plano-convex collection lens (E, f = 25.4 mm) and coupled into a trifurcated optical fiber bundle (D), which simultaneously feeds three synchronized StellarNet BLUE-Wave miniature spectrometers (Units 1–3) covering a combined spectral range of 397–1184 nm. Spectral acquisition is synchronized to the laser pulse via a TTL trigger signal derived from the Q-Switch Sync OUT output.

All spectral data were acquired using a custom-built LIBS system constructed on an optical breadboard to ensure high optomechanical stability and minimize vibrational interference (Figure 1).

The excitation source was a Q-switched, pulsed Nd:YAG laser (LOTIS TII, Minsk, Belarus; model LS-2134Y) operating at its second harmonic wavelength of 532 nm, producing pulses of 10–12 ns duration (Full Width at Half Maximum, FWHM) at a repetition rate of 15 Hz. The pump energy was set to 18 J per flash, yielding an output energy of approximately 120 mJ per pulse. The beam was directed onto the sample surface by a plano-convex lens (focal length: 75 mm, N-BK7, LA1608; Thorlabs, Newton, NJ, USA) to achieve the irradiance required for consistent plasma generation. The tissue sample was mounted on a manual XYZ positioning stage (Thorlabs, NJ, USA) to allow precise targeting.

Plasma emission was collected at 45° relative to the incident laser beam to minimize interference from laser scattering and specular reflection. The collection optics comprised a plano-convex lens (focal length: 25.4 mm, N-BK7, LA1951; Thorlabs), oriented with its flat face toward the sample, that focused the plasma plume emission onto the input aperture of a trifurcated optical fiber bundle, transmitting the collected light simultaneously to three spectrometer channels. The potential residual laser beam was safely terminated by a beam dump (LOTIS TII, Minsk, Belarus) positioned behind the sample stage. Spectral acquisition was synchronized to the laser pulse via the Q-Switch Sync OUT signal, which was conditioned through a voltage divider circuit to provide a TTL-compatible trigger (2.0–3.9 V logic high) to the spectrometer acquisition system.

The multi-channel detection system comprised three synchronized miniature spectrometers (StellarNet BLUE-Wave series; StellarNet, Inc., Tampa, FL, USA), each configured with a different diffraction grating to optimize spectral resolution across a specific sub-range: Unit 1-BLUE-Wave LSR-VIS4-14, 2400 g/mm grating; Unit 2-BLUE-Wave LSR-VIS4b-14, 1800 g/mm grating; and Unit 3-BLUE-Wave LSR-NIR3b-14, 1200 g/mm grating. The combined system provided continuous spectral coverage from approximately 397 to 1184 nm across 6141 wavelength channels, with a manufacturer-specified average optical resolution of approximately 0.1 nm. Each spectrometer utilized a 2048-element CCD linear array detector. Spectral acquisition was triggered by the Q-Switch synchronization signal with a software-configured trigger delay of approximately 200 µs; the CCD integration time per pulse was set to 1.05 ms. Because this integration window substantially exceeds the plasma lifetime (~100 µs for nanosecond LIBS), the acquired spectrum represents the temporally integrated plasma emission, including both continuum and atomic line contributions. To account for the initialization latency differences among the three spectrometer units (14.3 ms, 22.1 ms, and 29.3 ms respectively), the acquisition software was configured in episodic multi-channel mode with external trigger synchronization, ensuring simultaneous capture from all three channels. For each full-spectrum measurement point on a tissue sample, each of the three spectrometer channels independently wrote a raw data file representing the accumulation of 45 consecutive laser pulses over its corresponding wavelength sub-range; a complete measurement point therefore corresponds to three raw .EPX files (except for the Non-Tissue control class, whose acquisition software wrote a single, already channel-merged file per point), controlled by the acquisition software to improve the signal-to-noise ratio prior to computational processing. At the 15 Hz laser repetition rate, this corresponds to a per-point acquisition time of approximately 3 s; the spectral classification subsequently requires sub-millisecond inference time once the spectrum is acquired. The number of full-spectrum measurement points acquired per specimen ranged from 1 to 3 for Connective Muscle, 1 to 2 for Aorta and Small Intestine, and was fixed at 1 for Large Intestine, Nerve, and Non-Tissue, for a total of 94 spectra (250 files) across the 69 units of the dataset: 79 measurement points from 235 raw .EPX instrument files over the 54 biological specimens, plus the 15 synthetic Non-Tissue records (Section 2.1). At three channel files per point, the 79 biological measurement points would nominally amount to 237 raw .EPX files; the actual count is 235 because, for one measurement point of a Connective Muscle specimen, only the Unit 1 file (397.24–598.38 nm) was written to disk, the Unit 2 and Unit 3 files having been lost at acquisition time. That specimen contributed two further measurement points for which all three channel files are present, so its consolidated spectrum still spans the full 397–1184 nm range and no reported result is affected by the two missing files.

Figure 1 shows the custom-built LIBS experimental setup assembled on an optical breadboard. The left panel details the sample stage and focusing optics, identifying the main optical components: the focusing lens (A), manual XYZ sample positioning stage (B), beam dump (C), and the plasma collection assembly comprising the collection lens and the trifurcated optical fiber coupler (D, E). The right panel shows the three synchronized StellarNet BLUE-Wave miniature spectrometers connected to the fiber bundle, each covering a distinct spectral sub-range and collectively providing continuous coverage from 397 nm to 1184 nm.

### 2.3. Spectral Data Preprocessing and Aggregation

#### 2.3.1. ‘Peak Max over Baseline’ Aggregation

**Figure 2:**
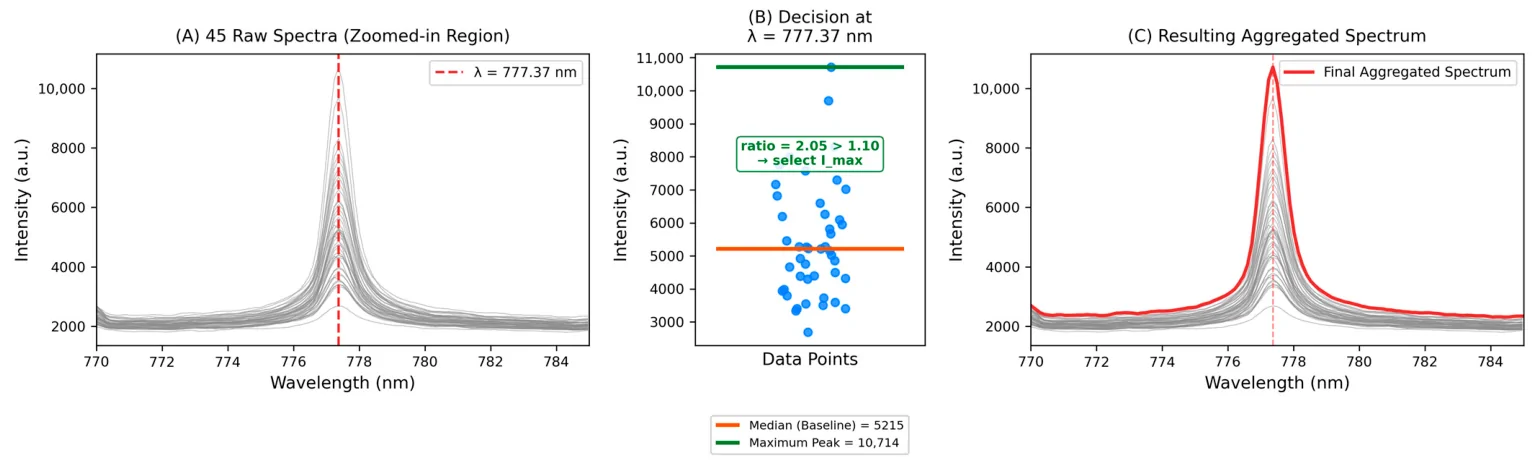
Visual illustration of the ‘peak max over baseline’ spectral aggregation method applied to a representative Aorta specimen. (**A**) A zoomed spectral region (770–785 nm) of the 45 raw single-shot spectra from a single measurement point, showing inherent shot-to-shot intensity variability and the emergence of emission peaks above the spectral continuum. (**B**) Distribution of the 45 intensity values at λ = 777.37 nm, an emission peak within the O I atomic emission triplet (∼777 nm). (**C**) The final single aggregated spectrum (red line) overlaid on the raw data cloud, demonstrating suppression of shot noise in the continuum regions and retention of the genuine emission peaks. Grey lines in panels (**A**,**C**) are the 45 individual single-shot spectra; blue markers in panel (**B**) are the 45 intensity values recorded at that wavelength.

The raw output consisted of .EPX files, each containing a matrix of 45 individual single-shot spectra from one measurement point. A non-linear aggregation algorithm, ‘peak max over baseline’, was applied to distill this matrix into a single robust representative spectrum, amplifying genuine atomic emission peaks while suppressing stochastic background noise (Figure 2).

The algorithm operates on a per-wavelength basis. For each discrete wavelength *λ_i_*, the vector of 45 intensity values was extracted. The median of this vector (*I_median_*) was computed as a robust estimator of the local baseline signal. The maximum intensity value (*I_maz_*) was then identified. A conditional assignment rule was applied: if *I_maz_* exceeded the baseline by more than a threshold of 10% (*I_max_* > 1.1 × *I_median_*), the maximum value was retained as the definitive intensity for that wavelength, indicating the presence of an emission peak above the noise floor. Otherwise, the more stable median value was assigned. This non-linear filtering was iterated across all wavelength variables, transforming the 45-spectrum matrix into a single aggregated spectrum vector per measurement point. The 10% threshold was selected empirically based on the observed shot-to-shot variability in background regions; sensitivity to this threshold was verified to be low within the range 5–20%. The sensitivity of this 10% threshold was assessed by repeating the complete consolidation and Extra Trees + Base classification pipeline at thresholds of 5%, 10%, 15% and 20% over the same ten validation repetitions, yielding weighted F1-scores of 0.941 ± 0.086, 0.934 ± 0.118, 0.927 ± 0.116 and 0.934 ± 0.118, respectively. Performance therefore remains within a narrow band across the whole range, confirming that the classification outcome is not sensitive to the precise value of this parameter.

Figure 2 illustrates the ‘peak max over baseline’ aggregation method applied to a representative Aorta specimen. Panel (A) displays the 45 raw single-shot spectra in the 770–785 nm window, showing the shot-to-shot intensity variability characteristic of nanosecond LIBS and the emergence of emission peaks above the spectral continuum. Panel (B) shows the intensity distribution at λ = 777.37 nm, a peak within the O I atomic emission triplet (∼777 nm): the baseline I_median_ = 5215 a.u. and the peak I_max_ = 10,714 a.u. yield a ratio of 2.05, substantially above the 1.10 threshold, so I_max_ is selected as the representative intensity at this wavelength. Panel (C) overlays the final aggregated spectrum (red line) on the raw data cloud, demonstrating effective suppression of shot noise in the continuum regions and retention of the genuine atomic emission peaks across the full spectral range.

#### 2.3.2. Dataset Construction

For each biological specimen, multiple spatial measurement points were acquired across the available tissue surface, with the number of measurement points per specimen determined by the physical dimensions of the sample. Each synthetic Non-Tissue record consists of a single full-spectrum entry, generated directly on the combined wavelength axis and therefore not following the three-channel file structure produced by the instrument, while biological tissue specimens yielded between 1 and 3 full-spectrum measurement points per specimen depending on tissue type and sample size; Large Intestine and Nerve specimens, being geometrically constrained, consistently yielded a single measurement point, whereas Connective Muscle, Aorta, and Small Intestine specimens, with larger usable surfaces, yielded up to 2–3. In total, 235 raw .EPX instrument files, corresponding to 79 full-spectrum measurement points (237 minus the two channel files missing for a single point, see Section 2.2), were processed across the 54 biological specimens; together with the 15 synthetic Non-Tissue records, this yields the 250 files and 94 spectra of the complete dataset. For each acquisition file, the ‘peak max over baseline’ aggregation described in Section 2.3.1 was applied to its 45 single-shot spectra; for biological tissue specimens, the three channel files sharing a given measurement point were then combined via a wavelength-wise maximum (Section 2.2) to yield one representative, full-spectrum aggregated spectrum per measurement point. The multiple per-point spectra from the same specimen were then consolidated into a single specimen-level spectrum by retaining, at each wavelength, the maximum intensity observed across all measurement points of that specimen. This wavelength-wise maximum further ensures that the final spectrum preserves the most prominent emission peaks observed across the entire sample surface, reducing sensitivity to spatial heterogeneity within the specimen. A master data matrix was then constructed by compiling the 69 consolidated specimen-level spectra. Each row corresponds to a single biological specimen; each column represents the consolidated intensity at a specific wavelength and thus constitutes an independent feature. The final dataset therefore contains 69 observations and 6141 features. Two metadata columns were appended: (i) a label column encoding the ground-truth tissue class for supervised learning, and (ii) a group_id column containing a unique identifier for the biological specimen. The group_id field is fundamental for the validation protocol described in Section 2.4, enabling strict specimen-level data separation during cross-validation.

### 2.4. Experimental Design and Validation Protocol

#### 2.4.1. Group-Based Data Partitioning (GroupShuffleSplit)

Standard K-fold cross-validation does not enforce specimen-level separation and permits data leakage in nested biomedical datasets. To prevent this, the GroupShuffleSplit algorithm was employed, partitioning specimens (not individual spectra) into training (80%) and test (20%) sets. All spectra from each specimen were kept together, guaranteeing that no specimen’s data was distributed between training and test sets.

#### 2.4.2. Repeated Cross-Validation (*N* = 10)

The GroupShuffleSplit partitioning was repeated *N* = 10 times with different random states (seeds 0 through 9), generating 10 training–test set pairs. Because specimen assignment is redrawn at random in each repetition, the resulting test sets overlap: a given specimen may be tested in several repetitions. The ten results are therefore repeated outer test evaluations of the same dataset, and not ten mutually independent evaluations; the reported standard deviation measures sensitivity to training set composition rather than the dispersion of independent samples. Repeating the evaluation across multiple training data compositions (i) provides a comprehensive assessment of model stability across diverse training subsets and (ii) enables final performance metrics to be reported as mean ± standard deviation, quantifying sensitivity to training data variation and providing a statistically meaningful measure of generalization performance.

#### 2.4.3. Class Imbalance Correction

Within each of the 10 training–test iterations, the Synthetic Minority Oversampling Technique (SMOTE) [20] was applied exclusively to the training data to generate synthetic samples for under-represented tissue classes. The SMOTE k_neighbors hyperparameter was dynamically set to (n_minority-1), where n_minority is the number of samples in the smallest class of that specific training fold, ensuring algorithmic robustness across folds.

### 2.5. Machine Learning Framework

A systematic comparative analysis was conducted to identify the optimal combination of feature preprocessing and classification algorithm. All experiments were implemented in Python 3.13 using scikit-learn [21] and imbalanced-learn [22].

#### 2.5.1. Feature Preprocessing Pipelines

Three preprocessing strategies were evaluated. (1) Base: Standard z-score normalization (zero mean, unit variance) applied to all spectral features. (2) PCA: Z-score normalization followed by Principal Component Analysis (PCA) retaining the top 10 principal components. This number was chosen based on a preliminary variance analysis conducted on the full dataset prior to model evaluation, which showed that 10 components consistently captured more than 99% of the total spectral variance, a finding subsequently confirmed fold-by-fold (99.71 ± 0.03% across the 10 cross-validation repetitions). This aggressive yet near-lossless dimensionality reduction was intended to test whether the discriminative information is concentrated in a low-dimensional subspace. (3) RFE: Z-score normalization followed by Recursive Feature Elimination (RFE) using a Decision Tree estimator to select the 30 most discriminative wavelength features. The target of 30 features was chosen to represent a ratio of approximately 2 features per tissue class, consistent with commonly used heuristics for high-dimensional spectral datasets where highly correlated adjacent wavelengths are expected.

#### 2.5.2. Classification Algorithms

Ten diverse classification algorithms were evaluated: Logistic Regression (LR), Random Forest (RF), Support Vector Machine with RBF kernel (SVM), K-Nearest Neighbors (KNN), Decision Tree (DT), Gradient Boosting (GB), Gaussian Naive Bayes (NB), Multi-layer Perceptron (MLP), Extra Trees (ET), and AdaBoost (AB).

### 2.6. Hyperparameter Optimization and Performance Evaluation

For each of the 30 algorithm-pipeline configurations, hyperparameters were optimized within each outer cross-validation fold using an inner GridSearchCV with StratifiedKFold (5 splits, shuffled, random_state = 42) applied exclusively to the training data. It is noted that this inner StratifiedKFold does not enforce specimen-level separation within the training fold; a StratifiedGroupKFold applied to the inner loop would be the fully group-aware alternative. However, since the inner loop serves only for hyperparameter selection (not for final performance estimation) and the training fold already excludes all test specimens, the expected impact of this limitation on final reported metrics is small. The optimization criterion was the weighted F1-score. The complete hyperparameter search grid for all 10 classifiers is reported in Table S1 (Supplementary Material). In total, 59 hyperparameter configurations were evaluated per pipeline per outer fold, yielding 8850 inner model fits across the full experiment. Representative grid values include regularization strength C ∈ {1, 10, 100} and kernel coefficient γ ∈ {‘scale’, ‘auto’} for SVM, and number of estimators ∈ {100, 200} and maximum depth ∈ {10, 20, None} for ET/RF. Upon identifying the optimal hyperparameter set, the model was retrained on the full training fold and evaluated once on the corresponding, completely untouched test set. Final reported metrics, mean weighted F1-score and mean accuracy, plus their standard deviations, were computed across the 10 repeated outer test evaluations. Accuracy is reported as standard (unweighted) accuracy to facilitate comparison with the literature. Because all 30 configurations were evaluated on this same set of ten outer splits and the configuration reported as best was identified after inspecting those results, the figure reported for the winning configuration is an optimistic estimate of its generalization performance; this is discussed in Section 4.4.

## 3. Results

### 3.1. Final Dataset Composition

**Figure 3:**
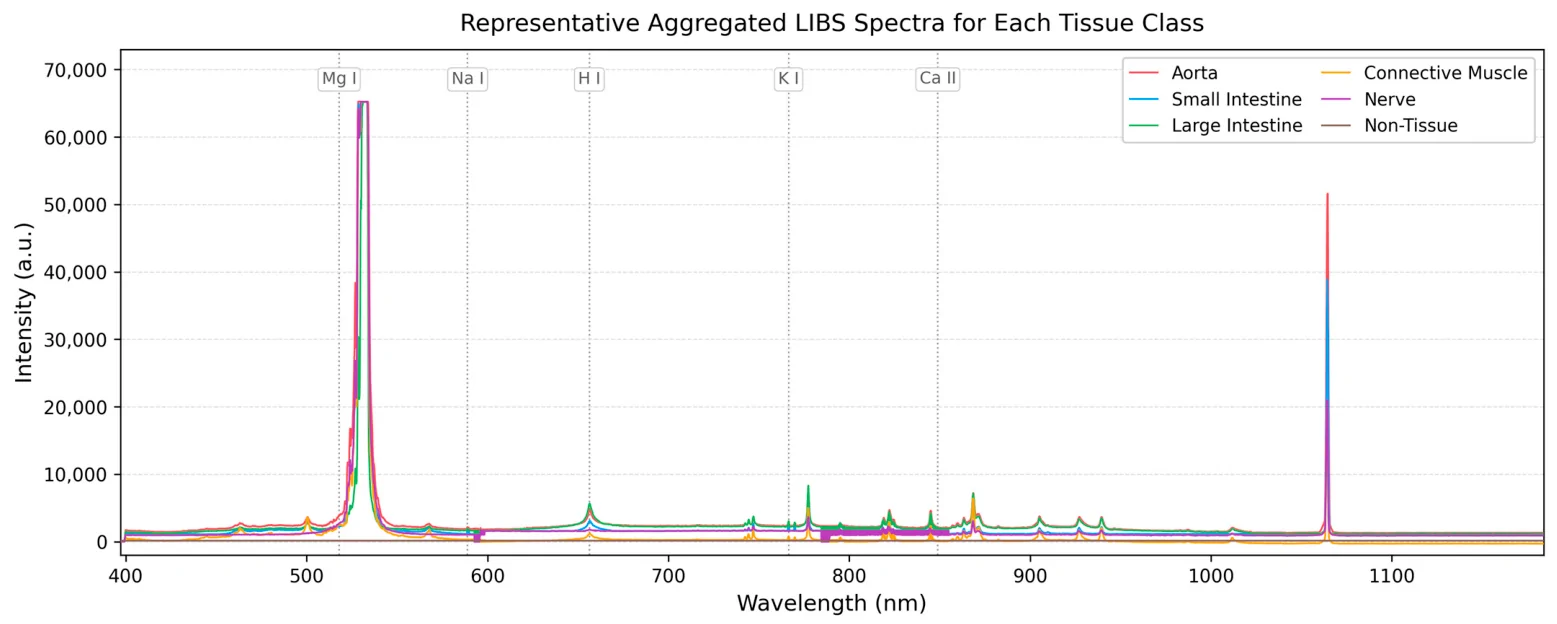
Representative aggregated LIBS spectra for each of the five biological tissue classes and for the synthetic Non-Tissue control, following ‘peak max over baseline’ preprocessing. Each spectrum displays distinct atomic emission peaks and intensity variations across the 397–1184 nm spectral range, which constitute the feature basis for classification. Prominent spectral regions correspond to Mg I (518 nm), Na I (589 nm), K I (766 nm), Ca II (849 nm), and C_2_ Swan band emissions. The Non-Tissue trace is the synthetic noise control described in Section 2.1, not a measured spectrum.

The spectral aggregation and curation pipeline yielded a final dataset of 69 unique biological specimens. The distribution across the five biological tissue classes and the synthetic Non-Tissue control is summarized in Table 1, which reveals a natural class imbalance: Connective Muscle is the most represented class (*n* = 16) and Small Intestine the least (*n* = 6). This imbalance was systematically addressed during training using SMOTE (Section 2.4.3). Representative aggregated spectra for each tissue class are shown in Figure 3, covering the 397–1184 nm range. Both shared spectral features and class-specific intensity patterns are visible, particularly in regions associated with Ca II, Mg I, Na I, K I, H I atomic emission lines and C_2_ Swan band emissions. These qualitative differences in elemental composition constitute the spectral basis for machine learning classification.

Figure 3 shows the representative aggregated LIBS spectra for each of the five biological tissue classes and for the synthetic Non-Tissue control over the 397–1184 nm range. All classes share a common spectral architecture dominated by strong class-specific differences evident in the C_2_ Swan band region (490–565 nm), the H I Balmer-alpha line (656 nm), the Na I doublet (589 nm), and the K I resonance line (766 nm). The synthetic Non-Tissue control is visually distinct from all biological tissues, showing a flat noise floor with no emission features across the full spectral range, as follows from the way it was generated (Section 2.1). Among biological tissues, Aorta and Connective Muscle display the most prominent Ca II and Mg I features, while Nerve spectra show relatively lower overall intensity, consistent with their lower mineral content. These inter-class spectral differences, reflecting tissue-specific elemental compositions, constitute the discriminative features exploited by the machine learning classifiers.

### 3.2. Comparative Evaluation of Model Performance

The comprehensive performance evaluation of all 30 experimental configurations was conducted using the rigorous repeated cross-validation protocol. The mean and standard deviation of the weighted F1-score and accuracy, computed across 10 repeated outer test evaluations, are reported in Table 2, ranked in descending order by mean F1-score.

Table 2 shows that the Extra Trees classifier combined with the Base preprocessing pipeline is the overall best-performing configuration (F1 = 0.934 ± 0.118; accuracy = 0.943 ± 0.094), followed closely by Random Forest with the same preprocessing (F1 = 0.927 ± 0.116). The two tree-based ensemble methods occupy the top two positions by a notable margin over the remaining configurations.

To assess whether the inclusion of the trivially separable Non-Tissue control class inflates the reported aggregate performance, the four top-performing configurations were re-evaluated after excluding Non-Tissue entirely (both from training and test), using the same 10 GroupShuffleSplit repetitions restricted to the five biological tissue classes. Table 3 reports the corresponding weighted F1-scores alongside the original six-class results.

As shown in Table 3, excluding the Non-Tissue class reduces the weighted F1-score by roughly 4–6 percentage points for the tree-based ensembles (Extra Trees, Random Forest) and for SVM + Base, confirming that the trivially separable Non-Tissue class inflates the six-class aggregate metric. Notably, the SVM Base = SVM PCA equivalence reported above does not hold in the five-class setting (0.879 vs. 0.915): the exact gamma-scale coincidence described in Section 3.2 is a property of the full six-class dataset’s variance structure and should not be interpreted as a general SVM/PCA identity. It should also be noted that the ranking of the four configurations is not preserved between the two tasks: Extra Trees + Base, the best configuration on the six-class problem, becomes the weakest of the four in the five-class setting (0.875 ± 0.096 against 0.879 ± 0.097 for Random Forest + Base, 0.879 ± 0.102 for SVM + Base and 0.915 ± 0.118 for SVM + PCA). The differences are small relative to the fold-level standard deviations, and we do not claim that any one configuration is superior on the biological task; the instability of the ranking is itself evidence that the choice of a single best configuration should be treated with caution (Section 4.4).

To characterize per-class discriminability, the mean normalized confusion matrix for the best-performing configuration (Extra Trees + Base), averaged across the 10 cross-validation folds, is reported in Table 4, and per-class precision, recall, and F1-score are summarized in Table 5.

As shown in Table 4 and Table 5, the per-class analysis reveals that three of the five biological classes—Aorta, Connective Muscle and Nerve—are classified with high mean recall (≥0.90), as is the synthetic Non-Tissue control. The primary source of classification error is the confusion between Small Intestine and Large Intestine (29.2% of Small Intestine specimens misclassified as Large Intestine), which is biologically expected given the shared anatomical origin, similar wall composition, and overlapping elemental fingerprints of the two intestinal tissue types. The low per-class support for Small Intestine (present in only 6 of the 10 folds, with a mean of 1.8 specimens per fold when present) limits the reliability of its per-class statistics and is the primary driver of the high aggregate standard deviation; Nerve was likewise present in only 9 of the 10 folds. Across all 10 repetitions, 63 of the 69 specimens (91.3%) appeared in the test set at least once, with per-class coverage ranging from 100% (Small Intestine, Connective Muscle) to 75% (Nerve). To confirm that the weighted F1-score is not driven disproportionately by the larger classes, macro-averaged F1 and balanced accuracy were also computed for the best-performing configuration: macro-F1 = 0.912 ± 0.125 and balanced accuracy = 0.948 ± 0.076 for Extra Trees + Base, both closely tracking the weighted metrics reported above. As a complement to the standard deviation, bootstrap 95% confidence intervals (10,000 resamples over the 10 fold-level scores) for the weighted F1-score are [0.856, 0.993] for Extra Trees + Base, [0.849, 0.984] for Random Forest + Base, and [0.876, 0.957] for SVM + Base; these are approximate rather than strict frequentist intervals, since the 10 repeated GroupShuffleSplit folds are not fully independent. The Non-Tissue control class is classified perfectly across all folds, which follows from its construction as featureless Gaussian noise (Section 2.1) and not from any measured property of a background surface.

A noteworthy result is that the SVM (RBF) classifier achieved numerically identical performance under both the Base and PCA (10 components) preprocessing pipelines (F1 = 0.917 ± 0.069, with fold-level scores identical across all 10 repetitions). This result has been verified against the original analysis code and is confirmed to be a genuine scientific finding, not an implementation artifact. The underlying mechanism is as follows: after StandardScaler normalization, the gamma = ‘scale’ parameter of the RBF kernel is computed as γ = 1/(*p* × σ^2^), where *p* is the number of features and σ^2^ is the mean per-feature variance. For the Base pipeline (*p* = 6141, σ^2^ ≈ 1 per standardized feature), γ_base = 1/6141 ≈ 1.63 × 10^−4^. For the PCA pipeline (*p* = 10 components, each with mean variance ≈ 612), γ_pca = 1/(10 × 612) ≈ 1.63 × 10^−4^—virtually identical. This invariance arises because the 10 leading principal components capture 99.71 ± 0.03% of the total spectral variance across all folds, so the product *p* × σ^2^ is conserved under the PCA transformation. Consequently, both pipelines present the SVM with equivalent kernel scales, and the near-lossless compression achieved by the 10-component PCA results in functionally equivalent decision boundaries across all validation partitions. This equivalence is, however, a property of the specific six-class dataset composition rather than a general SVM/PCA identity: it no longer holds when the Non-Tissue class is excluded (see Table 3).

A consistent finding across all top models is that dimensionality reduction (PCA) and feature selection (RFE) did not improve classification performance and were often detrimental. The most striking example is Extra Trees: applying PCA reduced its mean F1-score from 0.934 to 0.839, a decrease of approximately 10 percentage points. Analogous degradation was observed for Random Forest (0.927 → 0.834) and Gradient Boosting (0.865 → 0.774). The AdaBoost + RFE combination exhibited the most extreme instability in the entire experiment (F1 = 0.688 ± 0.232), with individual fold scores ranging from 0.233 to 1.000. This anomalous behavior is attributed to an unfavorable interaction between RFE’s greedy feature selection and AdaBoost’s sensitivity to noisy features on small training subsets—a pattern characteristic of sequential boosting methods when the feature selection step is poorly conditioned. These findings collectively suggest that the informative content of the aggregated spectra is broadly distributed across the full spectral range and is best exploited by algorithms capable of natively handling high-dimensional feature spaces.

### 3.3. Model Stability and Performance Distribution Analysis

**Figure 4:**
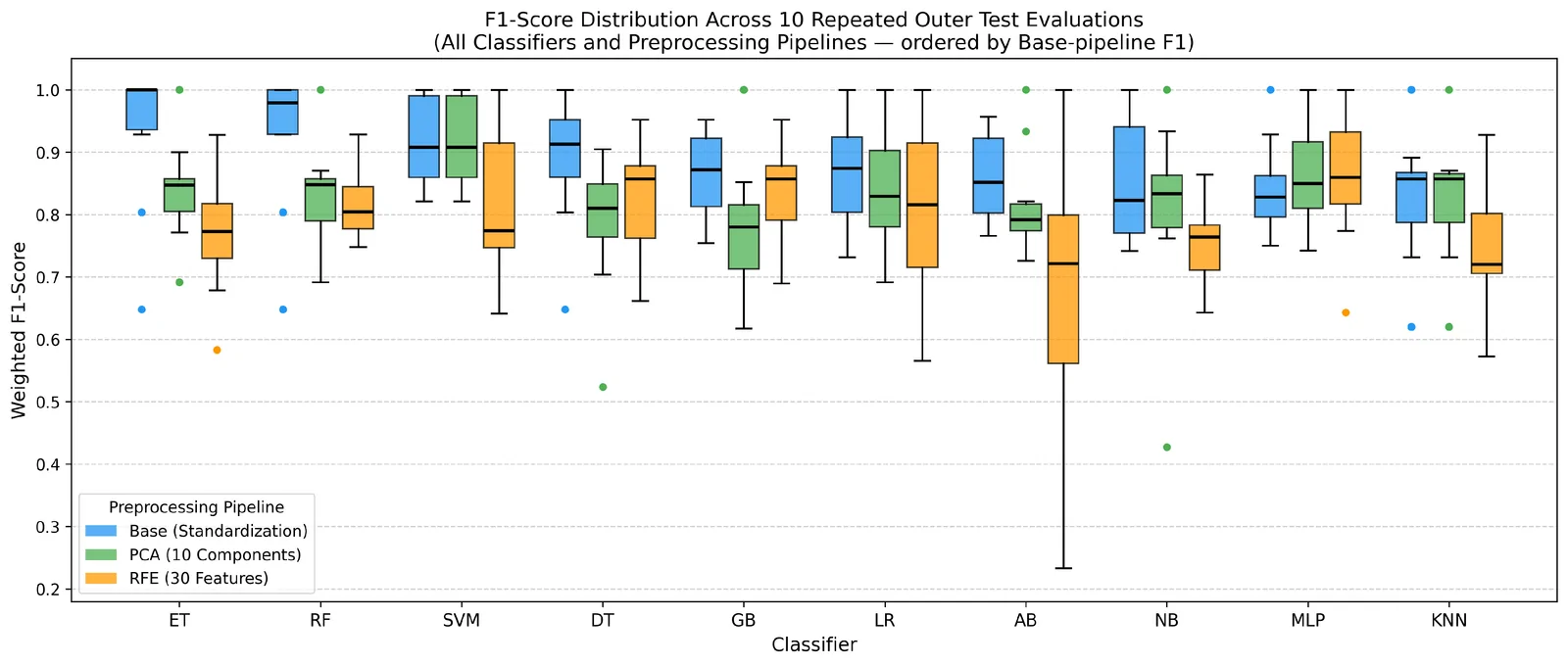
F1-score distribution across 10 validation repetitions for all 10 classifiers under each of the three preprocessing pipelines (blue: Base/Standardization; green: PCA/10 components; orange: RFE/30 features). Classifiers are ordered left to right by descending mean F1-score under the Base pipeline. The vertical axis starts at 0.18 so that the lower whisker of AdaBoost + RFE, whose worst fold reaches 0.233, is fully contained within the plotting area.

**Figure 5:**
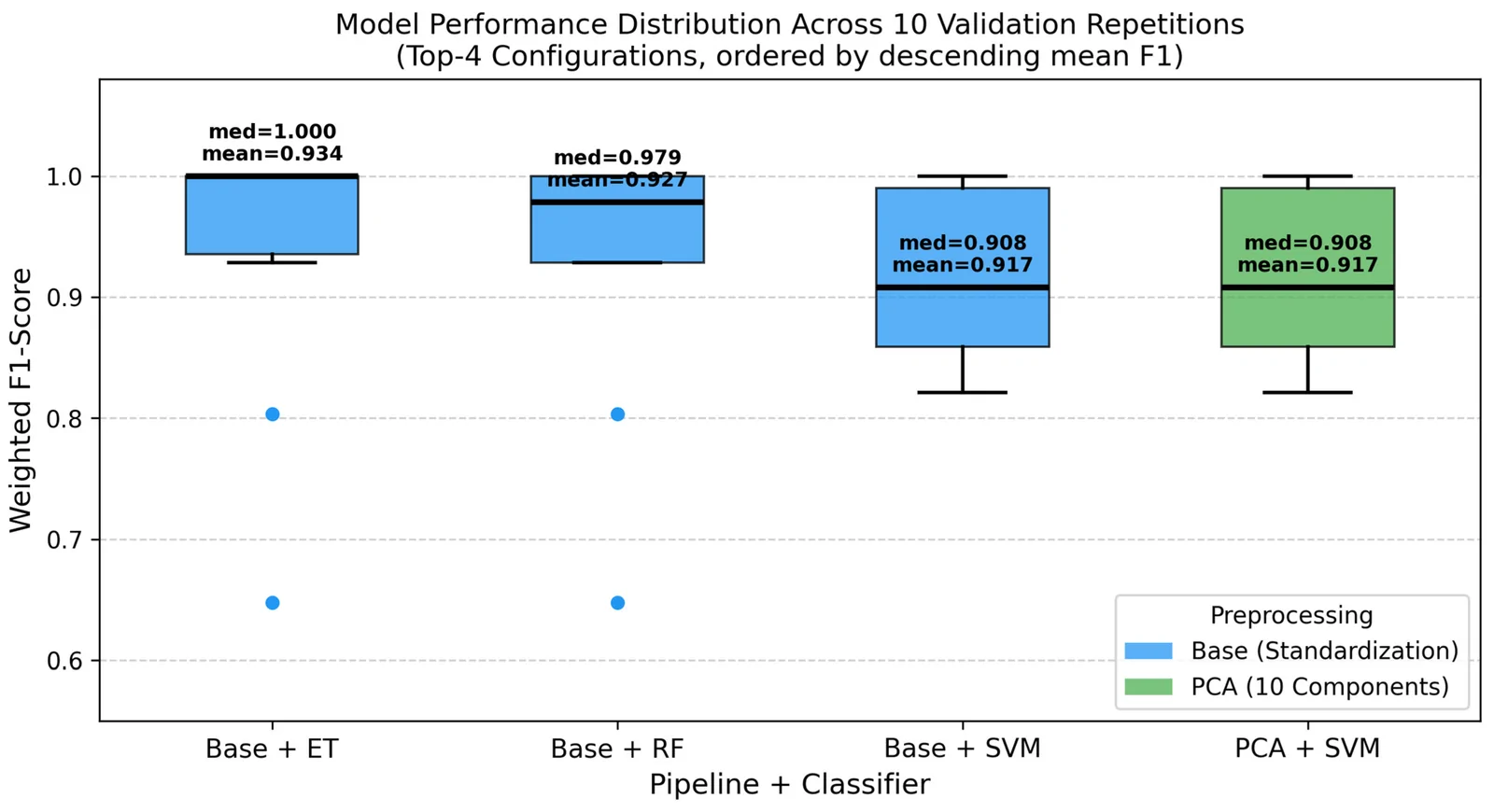
F1-score distribution across 10 validation repetitions for the four top-performing configurations. The center line denotes the median; box boundaries indicate the interquartile range (IQR); whiskers extend to 1.5 × IQR; circles represent outliers. Median and mean values are annotated above each box.

The distribution of F1-scores across the 10 repeated outer test evaluations for all classifiers and for the four highest-performing configurations is visualized in Figure 4 and Figure 5, respectively. These plots reveal a critical performance-versus-stability trade-off. Both Extra Trees and Random Forest exhibit relatively wide interquartile ranges and the presence of low-scoring outliers (F1 < 0.80 in some folds), indicating sensitivity to training set composition. In contrast, the SVM classifiers show compact distributions with small interquartile ranges and an absence of low-performance outliers, demonstrating markedly greater consistency across all 10 validation runs.

**Figure 6:**
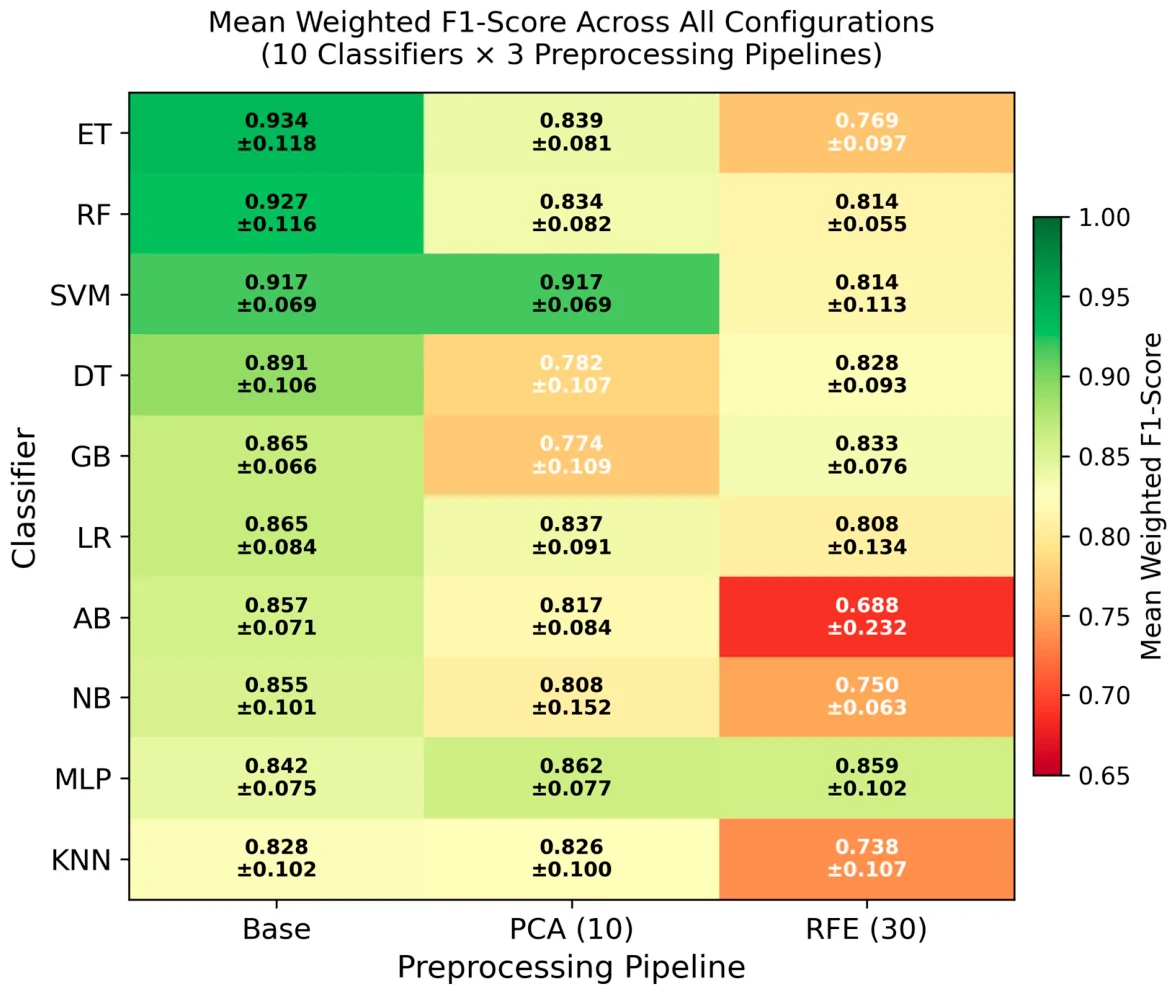
Heatmap of mean weighted F1-score (annotated with mean ± standard deviation) for all 30 experimental configurations (10 classifiers × 3 preprocessing pipelines). Classifiers are ordered by descending mean F1-score under the Base pipeline. Color scale ranges from red (low performance) to green (high performance). The Base pipeline consistently outperforms PCA and RFE reduction strategies across nearly all classifiers.

**Figure 7:**
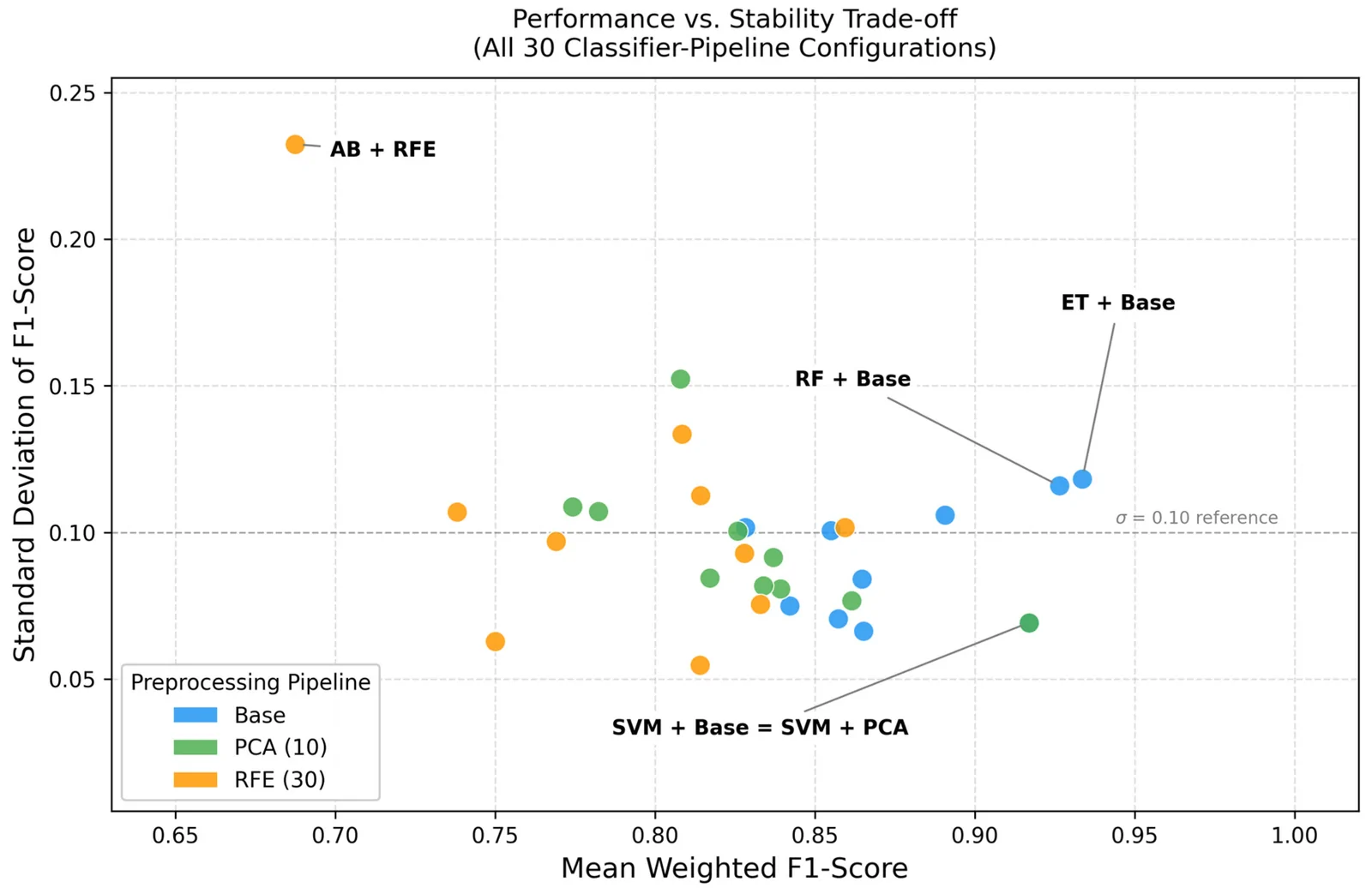
Performance versus stability trade-off for all 30 classifier-pipeline configurations. Each point represents one configuration; the horizontal axis shows the mean weighted F1-score and the vertical axis shows its standard deviation across 10 validation repetitions. Configurations in the lower-right quadrant (high mean, low variance) are most suitable for clinical deployment. The SVM classifiers (Base and PCA) uniquely combine high mean performance with low variability. Labels for the highlighted configurations are placed with leader lines to avoid overlap; SVM + Base and SVM + PCA share a single label because both yield identical values (0.917 ± 0.069). The mean F1-score and standard deviation of all 30 configurations are listed in Table 2.

**Figure 8:**
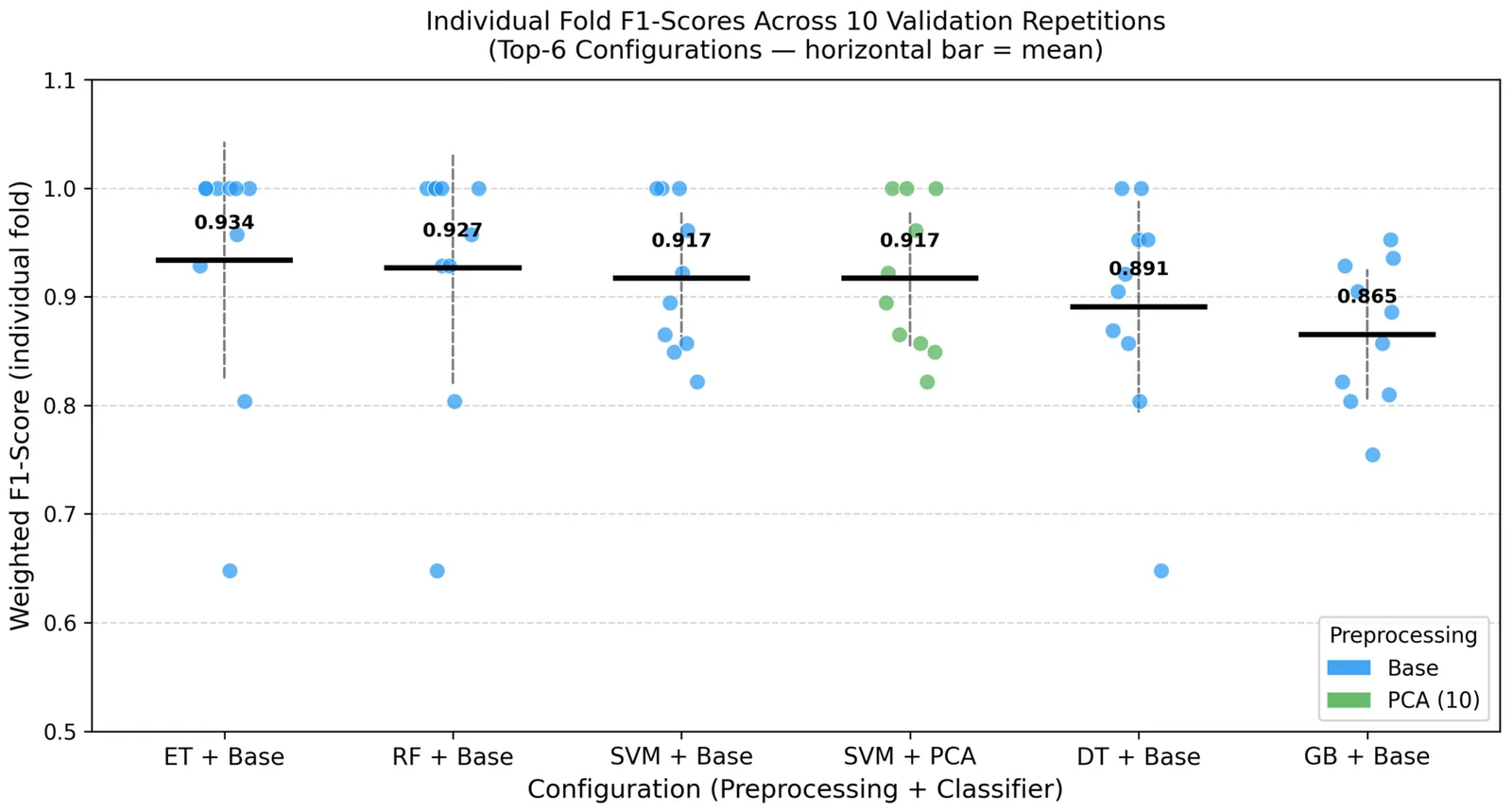
Individual F1-scores for the top 6 configurations across 10 repeated outer test evaluations. Each circle represents a single fold result; the horizontal black bar denotes the mean. ET + Base and RF + Base achieve the highest mean scores (0.934 and 0.927, respectively) but display a wider spread, whereas SVM + Base and SVM + PCA exhibit compact, consistent distributions, reflecting their superior reliability for clinical deployment.

Figure 4 provides an overview of the F1-score distributions for all 10 classifiers across the three preprocessing pipelines, confirming that the Base pipeline consistently outperforms PCA and RFE across nearly all classifiers. Figure 5 focuses on the four top-performing configurations, where the interquartile ranges and individual fold outcomes are compared in detail. In order to better analyze these results, and particularly the mean–standard deviation trade-off, Figure 6, Figure 7 and Figure 8 show more details. The heatmap in Figure 6 summarizes mean F1-score ± standard deviation for all 30 configurations simultaneously, highlighting the consistent superiority of the Base pipeline and the progressive performance degradation associated with PCA and RFE dimensionality reduction. Figure 7 plots mean F1-score against its standard deviation for each configuration, making the performance-versus-stability trade-off explicit: the SVM classifiers (Base and PCA) occupy the lower-right quadrant of high mean performance combined with low variability, identifying them as the most suitable candidates for clinical deployment. Figure 8 displays the individual fold-level F1-scores for the top 6 configurations, revealing the distribution spread: Extra Trees and Random Forest achieve the highest means (0.934 and 0.927, respectively) but exhibit a wider spread, while SVM maintains a compact, consistent distribution with no fold-level score below 0.821. Figure 5 is presented in descending order of mean F1 (Extra Trees, Random Forest, SVM + Base, SVM + PCA), consistent with the ordering used in Figure 6 and Figure 8. For Extra Trees + Base, the median F1-score across the 10 folds is 1.000 while the mean is 0.934; this reflects a right-skewed fold-score distribution in which the classifier scores perfectly in 6 of 10 folds but falls to between 0.648 and 0.957 in the remaining four, pulling the mean below the median. Where individual configuration labels in Figure 7 are difficult to distinguish due to close clustering, Table 2 provides the corresponding numerical values for all 30 configurations as a fallback reference.

These visualizations collectively reveal a clear performance-versus-stability trade-off. Both Extra Trees and Random Forest—despite their superior mean performance—exhibit relatively wide interquartile ranges. In fold 4 (where all test specimens belonged to a particularly challenging tissue composition), both classifiers recorded their minimum F1-score of 0.648, indicating meaningful sensitivity to the composition of the training set. The identical worst-case fold for both ensemble methods (fold 4, F1 = 0.648) further highlights the challenge posed by certain specimen groupings in this relatively small dataset. In stark contrast, the SVM classifier demonstrates a compact distribution with a small interquartile range and no extreme low-performance outliers, showing markedly greater consistency across all 10 validation runs.

## 4. Discussion

### 4.1. Interpretation of the Optimal Model and the Role of Preprocessing

The comparative analysis consistently identifies tree-based ensemble methods, Extra Trees and Random Forest, as the highest-performing classifiers for this task. This outcome is theoretically consistent with the intrinsic properties of these algorithms when applied to high-dimensional spectral data: both algorithms aggregate predictions from a large number of decorrelated decision trees, are inherently robust to uninformative variables through internal random feature subsampling, and are capable of capturing complex, non-linear interactions between spectral features without requiring a priori domain knowledge [23]. These properties make them well-suited to the *p* ≫ *n* setting characteristic of LIBS spectral datasets. The marginally superior mean performance of Extra Trees over Random Forest (F1: 0.934 vs. 0.927) is consistent with the prior literature on ensemble methods for spectroscopic data. Extra Trees introduces an additional layer of randomization by selecting both the split variable and the split threshold randomly, rather than optimizing the threshold at each node as in standard Random Forest. This increased stochasticity tends to reduce variance at a slight cost to bias, which can be advantageous in high-dimensional problems where overfitting risk is elevated. To formally assess whether this performance difference is statistically significant, a Wilcoxon signed-rank test was applied to the 10 paired fold-level F1-scores of ET + Base vs. RF + Base. Inspection of the fold-level data reveals that the two configurations produced identical F1-scores on 9 of the 10 validation folds; they differed only in fold 9 (ET: 1.000, RF: 0.929). The Wilcoxon signed-rank test, which discards tied pairs, therefore operates on a single effective pair (*n* = 1), yielding W = 0 and *p* = 1.000. With only one effective non-tied pair, this test is underpowered, and its non-significant result should not be interpreted as formal proof of equivalence between the two configurations—only as an absence of evidence for a difference, which is a weaker claim. Nonetheless, the observed mean difference of 0.007 F1-score is attributable entirely to a single fold outcome, and the two configurations are practically comparable based on their close point estimates and near-identical fold-by-fold behavior. Both models should therefore be regarded as equally strong candidates for further development.

The per-class analysis (Table 4 and Table 5) provides clinically relevant insight into the sources of classification error. Three of the five biological classes, Aorta, Connective Muscle and Nerve, together with the synthetic Non-Tissue control, are classified with perfect or near-perfect recall across all folds; for these three biological classes, this demonstrates that their LIBS elemental fingerprints are robustly discriminable by the ensemble model, whereas the synthetic Non-Tissue control is separable by construction (Section 2.1). The only meaningful source of error is the confusion between Small Intestine and Large Intestine (29.2% misclassification rate), which is biologically expected: both tissues derive from the same embryological origin and share similar wall architecture, smooth muscle composition, and electrolyte profiles, leading to overlapping LIBS spectral signatures. From a clinical perspective, this confusion has limited intraoperative impact in scenarios where the surgeon already knows they are operating in the intestinal region; in such contexts, the five-class discrimination (excluding the inter-intestinal confusion) would be clinically actionable. Expanding the dataset to include more Small Intestine specimens, the most under-represented class with only six specimens, is the primary recommended step to improve this class-specific performance. A related, complementary five-class analysis excluding the trivially separable Non-Tissue class entirely (Table 3) shows a 4–6 percentage point reduction in weighted F1 for the tree-based ensembles, confirming that part of the six-class aggregate performance is attributable to the ease of separating a featureless synthetic control from biological tissue rather than to discrimination among tissue types themselves.

A particularly illuminating finding was the consistent failure of dimensionality reduction (PCA) and feature selection (RFE) to improve the performance of top-tier models, in many cases causing substantial degradation. We attribute this to the ‘peak max over baseline’ aggregation strategy acting as an implicit, physics-informed feature extraction step: by suppressing stochastic background noise and retaining only emission peaks exceeding the significance threshold, it preprocesses spectra to highlight the most discriminatory elemental information. The full aggregated spectral profile therefore encodes meaningful classification information broadly distributed across multiple wavelength regions, and aggressive dimensionality reduction risks discarding diagnostically relevant features. Notably, the SVM result confirms that the aggregated spectra have a strongly low-dimensional intrinsic structure: 99.71 ± 0.03% of the total variance is captured by just 10 principal components (out of 6141 wavelength features), confirming the effectiveness of the aggregation in concentrating discriminative information into a compact spectral manifold.

### 4.2. Performance Versus Stability: Clinical Translation Perspective

While Extra Trees and Random Forest delivered the highest mean F1-scores, their per-fold performance distributions reveal an important limitation for clinical applications: individual fold F1-scores as low as 0.648 were observed (fold 4 for both ET and RF), indicating non-negligible sensitivity to the specific composition of the training set. With only 69 specimens in the dataset, the composition of the 20% test set (approximately 14 specimens) can vary considerably across repetitions, and certain specimen groupings represent more challenging classification scenarios than others.

In contrast, the SVM classifier, despite a marginally lower mean F1 = 0.917, exhibited a substantially more compact performance distribution with a small interquartile range and an absence of extreme low-performance outliers across all 10 validation runs. This indicates that SVM’s performance is robust and predictable across diverse training data subsets. From a purely accuracy-maximizing perspective, Extra Trees and Random Forest are the preferred models. However, the performance-versus-reliability trade-off is a paramount consideration for clinical translation. For high-stakes applications such as intraoperative tissue identification during guided surgery, where an erroneous classification could directly affect a surgical decision, a model with predictable, bounded performance and a well-characterized worst-case scenario may be preferable to one with a higher mean that carries a risk of severe degradation [24]. This study therefore provides evidence for two distinct clinical profiles: Extra Trees and Random Forest as the highest-mean-performance models suitable for controlled settings, and SVM as the more suitable choice where regulatory predictability and consistent performance under varied training conditions are prioritized.

Critically, intraoperative deployment requires not only high classification accuracy but also acceptable total response latency. It is important to distinguish two separate time scales: (i) the per-laser-pulse plasma emission acquisition time, which is governed by the CCD integration window (1.05 ms) and is therefore sub-millisecond in terms of data content; and (ii) the per-classification-event acquisition time, which in the present configuration spans approximately 3 s (45 pulses at 15 Hz). The trained classifier itself adds sub-millisecond inference latency once the spectrum is assembled. Whether 3 s of spectral acquisition is compatible with intraoperative workflows depends on the surgical modality and the rate of tissue transitions at the ablation interface. Future work should evaluate the minimum number of pulses required to maintain acceptable classification performance, potentially reducing acquisition time substantially, and integrate the pipeline with the acquisition software to measure end-to-end system latency under realistic operating conditions. In this direction, we additionally verified that classification does not require specimen-level spectral aggregation: re-training Extra Trees + Base on individual, full-spectrum measurement points (94 points across the 69 specimens, with GroupShuffleSplit still enforcing specimen-level train/test separation) yielded a weighted F1-score of 0.944 ± 0.058, comparable to and slightly more stable than the specimen-level result. This supports the feasibility of classifying a single measurement point in real time, without waiting to acquire and aggregate multiple points across the full tissue surface.

### 4.3. The Critical Importance of Group-Based Validation

The most important methodological contribution of this work is the empirical demonstration of why group-based validation is non-negotiable for nested biomedical spectral datasets. Under standard K-fold splits that ignore specimen grouping, spectra from the same biological specimen can appear in both training and test sets, producing inflated estimates that do not reflect true generalization to a new, unseen specimen. By enforcing strict specimen-level separation and repeating the evaluation 10 times, our protocol yields performance estimates with quantified uncertainty: for our best model, a mean accuracy of 94.3% ± 9.4%. The standard deviation itself is a critical result, informing practitioners about the expected range of performance under real-world deployment conditions [19]. An important caveat, discussed further in Section 4.4, is that our group_id was assigned at the level of each dissected tissue specimen rather than at the level of the source animal: with the 69 specimens contributed by five source animals, and no reliable record of which specimens share a common animal of origin, our protocol rigorously prevents leakage between tissue pieces but does not guarantee separation at the animal level.

### 4.4. Limitations and Future Directions

The dataset was sourced from a single species (bovine) and acquired on a single, custom-built LIBS instrument. While ex vivo bovine tissue provides a controlled and ethically accessible model, direct translation to human tissue or in vivo scenarios cannot be assumed without further validation. The definitive next step is an external validation study applying the trained model, without re-training, to an independent dataset acquired from a different species, instrument, or laboratory.

A related and more fundamental limitation concerns the biological independence of the 69 specimens. Our group_id, used to enforce specimen-level separation during GroupShuffleSplit, was assigned per dissected tissue specimen, not per source animal. The 69 specimens were obtained from five bovine animals in total, collected during two visits to the abattoir (two animals in the first visit and three in the second), with the same animal in some cases contributing more than one specimen within a tissue class (for example, two sub-samples dissected from a single aorta). Our cross-validation protocol rigorously prevents spectra from the same tissue piece from being split across training and test sets but does not guarantee that tissue from the same source animal is fully held out between training and test. Taken together with the use of a single instrument, the absence of any external or human tissue validation, and the model selection procedure described below, this means that the present study is best characterized as an exploratory internal-validation study rather than a clinical validation. We regard this as a major limitation of the present work to be addressed in future studies through explicit animal-level tracking at collection time, and we report it here in full rather than understating it.

A second methodological limitation concerns the inclusion of the synthetic Non-Tissue control class (*n* = 15 simulated null-signal records, Section 2.1) in the six-class problem. Because these records consist of Gaussian noise and contain no emission features, they are separable from biological tissue by construction rather than through any measured physical property of a background surface, and the six-class aggregate metric is correspondingly optimistic. Its inclusion may marginally inflate the reported aggregate weighted F1-score relative to the five-class biological discrimination task. Table 3 (Section 3.2) reports this five-class analysis directly: excluding Non-Tissue reduces the weighted F1-score by roughly 4–6 percentage points for the tree-based ensembles and for SVM + Base, confirming this expectation quantitatively. Accordingly, the five-class figures in Table 3, and not the six-class aggregate, should be taken as the reference performance for discrimination among biological tissues. We note that a measured background class, acquired on the actual sample substrate, would be a more informative control and is a straightforward addition to future data collection campaigns.

A third limitation relates to the hyperparameter optimization step: the inner GridSearchCV uses StratifiedKFold, which does not enforce specimen-level separation within the training fold. While the expected impact on final performance estimates is considered small (the inner loop affects only hyperparameter selection, not final evaluation), this represents a deviation from the fully group-aware methodology advocated by this work. A related deviation concerns class-imbalance correction: SMOTE oversampling, together with the Base/PCA/RFE preprocessing steps, was fit once on the full outer-training fold rather than being re-fit within each inner cross-validation fold via a single combined pipeline. Re-running Extra Trees and Random Forest with SMOTE properly nested inside the inner GridSearchCV loop yielded weighted F1-scores of 0.926 ± 0.116 and 0.919 ± 0.113, respectively—within one percentage point of the original, non-nested results—confirming that this deviation has limited practical impact. We also re-ran the outer validation using StratifiedGroupKFold in place of GroupShuffleSplit: this achieved at least one test specimen of every class in 8 of the 10 folds (versus several folds lacking Small Intestine specimens under the original protocol), with comparable and slightly more stable performance (Extra Trees: F1 = 0.923 ± 0.064). Future implementations should adopt StratifiedGroupKFold together with a fully nested imblearn pipeline as standard practice.

A further limitation concerns model selection itself. The 30 classifier–pipeline configurations were all evaluated on the same ten outer splits, and the configuration reported here as best was chosen after those results were available. No outer partition was therefore held out from the selection step, and the weighted F1-score reported for the winning configuration is an optimistic estimate of the performance it would achieve on genuinely unseen specimens. An unbiased estimate would require either an additional untouched test set or a nested selection loop in which the comparison among configurations is repeated inside each outer fold. Since the leading configurations are separated by less than one percentage point of mean F1 (Table 2), the ranking among the top models should be read as indicative rather than definitive.

We further verified that the reported performance does not hinge on the choice of aggregation rule: re-consolidating the raw spectra with a simple mean, a median and a 10% trimmed mean in place of the ‘peak max over baseline’ rule yielded weighted F1-scores of 0.931 ± 0.081, 0.923 ± 0.114 and 0.923 ± 0.114, respectively, against 0.934 ± 0.118 for the rule adopted here, so the aggregation strategy is not the source of the reported performance. As a robustness check on the acquisition-count imbalance across tissue classes described in Section 2.3.2, we additionally re-ran Extra Trees + Base after capping every specimen to a single measurement point (matching the point count already fixed for Large Intestine, Nerve, and Non-Tissue): weighted F1 fell modestly from 0.934 ± 0.118 to 0.923 ± 0.064, indicating that the unequal point counts are a real but limited confounder rather than the dominant driver of the reported results. Several promising avenues exist for future work. First, the trained pipeline is ready for integration into real-time acquisition and classification software, enabling development of a prototype system for prospective intraoperative validation. Second, exploring deep learning architectures such as 1D Convolutional Neural Networks presents a compelling next step, as these could potentially learn hierarchical spectral features directly from minimally processed data. Finally, a systematic feature importance analysis from the Extra Trees model would identify the most discriminative atomic emission lines across tissue classes, enhancing model transparency and linking machine learning outputs back to underlying elemental spectroscopy.

Table 6 collects the primary analysis and every robustness analysis reported above in a single place, stating for each one the weighted F1-score obtained and the methodological concern it addresses. It is intended to prevent the sensitivity analyses from being read as competing results: the GroupShuffleSplit six-class figure is the primary analysis of this work, the five-class figure is the reference for biological tissue discrimination, and the remaining rows quantify how much the primary estimate moves when individual methodological choices are varied one at a time.

## 5. Conclusions

This study presented and rigorously evaluated an end-to-end machine learning pipeline for the automated classification of five biological tissue types, together with one synthetic null-signal control class, from LIBS spectral data, explicitly targeting the methodological requirements for intraoperative tissue identification in laser-guided surgery. By implementing a group-based repeated cross-validation protocol (GroupShuffleSplit, *N* = 10), we obtained performance estimates that account for the nested structure of biomedical spectral datasets and prevent leakage between spectra of the same dissected tissue specimen—a critical and often overlooked methodological requirement. The 69 specimens used in this study were obtained from five source animals; Section 4.4 discusses the resulting limits on independent biological replication that this implies.

The Extra Trees classifier coupled with standard feature normalization achieved the highest mean weighted F1-score of 0.934 ± 0.118 and accuracy of 0.943 ± 0.094, followed closely by Random Forest (F1 = 0.927 ± 0.116). A Wilcoxon signed-rank test on the 10 paired fold-level scores found no significant difference (W = 0, *p* = 1.000); however, the two models produced identical scores on 9 of 10 folds, leaving only one effective non-tied pair, so this result is underpowered and should be read as an absence of evidence for a difference rather than proof of formal equivalence. Both models nonetheless remain equally strong candidates for further development given their close point estimates. Per-class analysis revealed that three of the five biological classes, Aorta, Connective Muscle and Nerve, were classified with perfect or near-perfect recall, as was the synthetic Non-Tissue control, which is separable by construction, while the primary source of error was confusion between Small Intestine and Large Intestine (29.2% misclassification rate), attributable to their shared anatomical origin and overlapping elemental composition. Support Vector Machines, while yielding slightly lower average performance (F1 = 0.917 ± 0.069), demonstrated markedly greater stability across validation folds, with no fold-level score below 0.821, a critical property for clinical applications requiring predictable, bounded behavior. A consistent finding was that dimensionality reduction (PCA) and feature selection (RFE) did not improve performance and in some cases substantially degraded it, suggesting that the ‘peak max over baseline’ aggregation strategy effectively pre-extracts the relevant informative content and that the full processed spectrum is the optimal feature set for ensemble classifiers applied to LIBS tissue data.

These results, obtained from five source animals under controlled ex vivo conditions on a single custom-built instrument, establish a replicable methodological framework and a realistic internal performance benchmark for the development of LIBS-based tissue classification systems intended for guided surgery. Because the best of 30 configurations was selected on the same ten outer splits, the inner tuning loop was not group-aware, and neither external nor human tissue was tested, the present work should be read as an exploratory internal-validation study; the five-class figures of 0.875–0.915, rather than the six-class aggregate, is the appropriate reference for discrimination among biological tissues. Spectral acquisition currently requires approximately 3 s per measurement point while inference is sub-millisecond, so the system is properly described as a step toward real-time guidance rather than as a real-time system. Prospective validation in ex vivo human tissue, with animal-level tracking and an independent test set, is the immediate next step. This work also underlines the need for rigorous, specimen-separated validation protocols as a methodological standard in biomedical spectroscopy and machine learning research.

## Figures and Tables

**Table 1:** Distribution of the 69 dataset units by class: 54 biological specimens across five tissue classes, plus 15 synthetic Non-Tissue control records (Section 2.1).

Tissue Class	Number of Specimens
Aorta	11
Small Intestine	6
Large Intestine	13
Connective Muscle	16
Nerve	8
Non-Tissue (control)	15
Total	69

**Table 2:** Performance summary of all 30 experimental configurations (mean ± standard deviation across 10 cross-validation repetitions), ordered by descending mean weighted F1-score. Best result highlighted in bold.

Preprocessing	Classifier	F1-Score Mean ± SD	Accuracy Mean ± SD
**Base**	**Extra Trees**	**0.934 ± 0.118**	**0.943 ± 0.094**
Base	Random Forest	0.927 ± 0.116	0.936 ± 0.092
Base	SVM (RBF)	0.917 ± 0.069	0.914 ± 0.066
PCA (10)	SVM (RBF)	0.917 ± 0.069	0.914 ± 0.066
Base	Decision Tree	0.891 ± 0.106	0.893 ± 0.084
Base	Gradient Boosting	0.865 ± 0.066	0.864 ± 0.071
Base	Logistic Regression	0.865 ± 0.084	0.879 ± 0.076
PCA (10)	MLP	0.862 ± 0.077	0.871 ± 0.074
RFE (30)	MLP	0.859 ± 0.102	0.864 ± 0.098
Base	AdaBoost	0.857 ± 0.071	0.850 ± 0.071
Base	Naive Bayes	0.855 ± 0.101	0.857 ± 0.101
Base	MLP	0.842 ± 0.075	0.857 ± 0.067
PCA (10)	Extra Trees	0.839 ± 0.081	0.850 ± 0.079
PCA (10)	Logistic Regression	0.837 ± 0.091	0.850 ± 0.086
PCA (10)	Random Forest	0.834 ± 0.082	0.843 ± 0.074
RFE (30)	Gradient Boosting	0.833 ± 0.076	0.836 ± 0.059
Base	KNN	0.828 ± 0.102	0.836 ± 0.089
RFE (30)	Decision Tree	0.828 ± 0.093	0.829 ± 0.084
PCA (10)	KNN	0.826 ± 0.100	0.836 ± 0.089
PCA (10)	AdaBoost	0.817 ± 0.084	0.814 ± 0.096
RFE (30)	SVM (RBF)	0.814 ± 0.113	0.821 ± 0.108
RFE (30)	Random Forest	0.814 ± 0.055	0.814 ± 0.060
RFE (30)	Logistic Regression	0.808 ± 0.134	0.821 ± 0.113
PCA (10)	Naive Bayes	0.808 ± 0.152	0.814 ± 0.131
PCA (10)	Decision Tree	0.782 ± 0.107	0.793 ± 0.098
PCA (10)	Gradient Boosting	0.774 ± 0.109	0.771 ± 0.116
RFE (30)	Extra Trees	0.769 ± 0.097	0.779 ± 0.086
RFE (30)	Naive Bayes	0.750 ± 0.063	0.743 ± 0.069
RFE (30)	KNN	0.738 ± 0.107	0.757 ± 0.108
RFE (30)	AdaBoost	0.688 ± 0.232	0.707 ± 0.209

Base: z-score normalization only. PCA (10): normalization + PCA, 10 components. RFE (30): normalization + Recursive Feature Elimination, 30 features. SVM: Support Vector Machine with RBF kernel. MLP: Multi-Layer Perceptron. KNN: K-Nearest Neighbors.

**Table 3:** Weighted F1-score for the four top-performing configurations, comparing the original six-class task with a five-class task excluding the Non-Tissue control class (mean ± standard deviation across the same 10 cross-validation repetitions).

Classifier	Preprocessing	Weighted F1 (6 Classes)	Weighted F1 (5 Classes, No Non-Tissue)
Extra Trees	Base	0.934 ± 0.118	0.875 ± 0.096
Random Forest	Base	0.927 ± 0.116	0.879 ± 0.097
SVM (RBF)	Base	0.917 ± 0.069	0.879 ± 0.102
SVM (RBF)	PCA (10)	0.917 ± 0.069	0.915 ± 0.118

**Table 4:** Mean normalized confusion matrix for Extra Trees + Base (10 cross-validation folds). Values represent the mean fraction of specimens of each true class (rows) classified into each predicted class (columns). Averages computed with nanmean to account for folds in which small classes yielded no test specimens (i.e., each cell is the mean of the per-fold row-normalized confusion matrix, computed only across folds in which that true class had at least one test specimen).

True\Pred	Aorta	Small Int.	Large Int.	Conn. Muscle	Nerve	Non-Tissue
Aorta	1.000	0.000	0.000	0.000	0.000	0.000
Small Int.	0.000	0.667	0.292	0.000	0.042	0.000
Large Int.	0.000	0.083	0.917	0.000	0.000	0.000
Conn. Muscle	0.000	0.000	0.000	1.000	0.000	0.000
Nerve	0.000	0.000	0.000	0.000	1.000	0.000
Non-Tissue	0.000	0.000	0.000	0.000	0.000	1.000

**Table 5:** Per-class mean precision, recall, and F1-score (± standard deviation) for Extra Trees + Base, computed via the same nanmean-across-present-folds convention as Table 4 (i.e., excluding folds in which a class had zero test specimens; the fraction of folds in which each class was present is reported in the Support column for Small Intestine and Nerve).

Tissue Class	Precision	Recall	F1-Score	Mean Support/Fold
Aorta	1.000 ± 0.000	1.000 ± 0.000	1.000 ± 0.000	1.9 ± 0.8
Small Intestine	0.583 ± 0.492	0.667 ± 0.516	0.611 ± 0.491	1.8 (6/10 folds)
Large Intestine	0.885 ± 0.243	0.917 ± 0.171	0.862 ± 0.196	2.2 ± 1.0
Connective Muscle	1.000 ± 0.000	1.000 ± 0.000	1.000 ± 0.000	4.9 ± 1.3
Nerve	0.944 ± 0.167	1.000 ± 0.000	0.963 ± 0.111	1.7 (9/10 folds)
Non-Tissue (control)	1.000 ± 0.000	1.000 ± 0.000	1.000 ± 0.000	2.4 ± 1.3

**Table 6:** Primary analysis and robustness analyses reported in this work, with the weighted F1-score of Extra Trees + Base in each case and the methodological concern that each analysis addresses. All figures are means ± standard deviation over the same ten outer test evaluations unless stated otherwise.

Analysis	Weighted F1-Score	Methodological Concern Addressed
Primary analysis: GroupShuffleSplit (*N* = 10), six classes, Extra Trees + Base	0.934 ± 0.118	Specimen-level separation between training and test sets; reference analysis of this work
Five-class task, synthetic Non-Tissue control excluded (Table 3)	0.875	Inflation of the aggregate metric by a trivially separable class; reference figure for biological tissue discrimination
StratifiedGroupKFold in place of GroupShuffleSplit	0.923 ± 0.064	Folds in which the smallest classes contributed no test specimen
SMOTE and preprocessing fully nested inside the inner loop	0.926 ± 0.116	Resampling and scaling fitted on the whole outer training fold
One measurement point per specimen	0.923 ± 0.064	Unequal number of measurement points across tissue classes
Aggregation by mean/median/10% trimmed mean	0.931 ± 0.081/0.923 ± 0.114/0.923 ± 0.114	Dependence of the result on the ‘peak max over baseline’ rule
Aggregation threshold at 5%/15%/20%	0.941 ± 0.086/0.927 ± 0.116/0.934 ± 0.118	Sensitivity to the 10% threshold adopted in Section 2.3.1
Classification of individual measurement points	0.944 ± 0.058	Relevance to single-point intraoperative use, without specimen-level aggregation

## Data Availability

The aggregated spectral dataset, the analysis code, the fold-level validation results and fold assignments used in this study are available from the corresponding author upon reasonable request.

## Supplementary Materials

The following supporting information can be downloaded at https://www.mdpi.com/article/10.3390/s26165020/s1, Table S1: Complete Hyperparameter Search Grid for All Classification Algorithms.
