# Supplementary material for: A Specimen-Separated Machine Learning Benchmark Toward Real-Time Tissue-Type Identification in Guided Surgery Using Ex Vivo Bovine Laser-Induced Breakdown Spectroscopy
Source: Sensors (Basel). 2026 Aug 7;26(16):5020. doi: 10.3390/s26165020 (PMC13517582; doi:10.3390/s26165020)
Supplement: Supplementary file 1 [file sensors-26-05020-s001.zip › sensors-4446688-supplementary.pdf]

sensors MDPI

Supplementary Materials

Table S1. Complete Hyperparameter Search Grid for All Classification Algorithms

All hyperparameters were optimized within each outer cross-validation fold using an inner GridSearchCV with StratifiedKFold (n_splits=5, shuffle=True, random_state=42), scored by weighted F1. All stochastic estimators used random_state=42. Parallel execution was enabled (n_jobs=−1).

Classifier	Hyperparameter	Values Evaluated	Total Configs
Logistic Regression (OvR)	Regularization strength C	0.1, 1.0, 10.0	3
Random Forest	n_estimators	100, 200	8
	max_depth	10, 20	
	min_samples_split	2, 5	
SVM (RBF kernel)	Regularization strength C	1, 10, 100	6
	Kernel coefficient γ	'scale', 'auto'	
K-Nearest Neighbors	n_neighbors	3, 5, 7	3
Decision Tree	max_depth	None, 5, 10, 20	12
	min_samples_split	2, 5, 10	
Gradient Boosting	n_estimators	100, 200	8
	max_depth per tree	3, 5	
	learning rate	0.05, 0.1	
Naive Bayes	var_smoothing	1e-9, 1e-7, 1e-5	3
MLP Neural Network	hidden_layer_sizes	(100,), (100, 50)	4
	L2 regularization (α)	0.0001, 0.001	
Extra Trees	n_estimators	100, 200	6
	max_depth	10, 20, None	
AdaBoost	n_estimators	50, 100, 200	6
	learning rate	0.5, 1.0	
		Total per pipeline per outer fold:	59

gamma='scale': $\gamma = 1/(n\_features \times Var[X])$, computed from training data at each fold. gamma='auto': $\gamma = 1/n\_features$. Logistic Regression used solver 'liblinear' wrapped in OneVsRestClassifier. SVM used probability=True. MLP used max_iter=2000. AdaBoost used algorithm='SAMME'. Total inner model fits: 59 configs × 5 folds × 3 pipelines × 10 repetitions = 8,850 fits.
